# Supplementary material for: DMRforPairs: identifying Differentially Methylated Regions between unique samples using array based methylation profiles
Source: BMC Bioinformatics. 2014 May 15;15:141. doi: 10.1186/1471-2105-15-141 (PMC4046028; doi:10.1186/1471-2105-15-141)
Supplement: Additional file 2 — DMRforPairs output for the comparison of A431-MCF7 and NA17018-NA17105. Please start from the HTML files in each folder. Available via the BMC Bioinformatics website. [file 1471-2105-15-141-S2.zip › 1394847754114233_MOESM2_ESM/A431_MCF7/figures/28057.pdf]

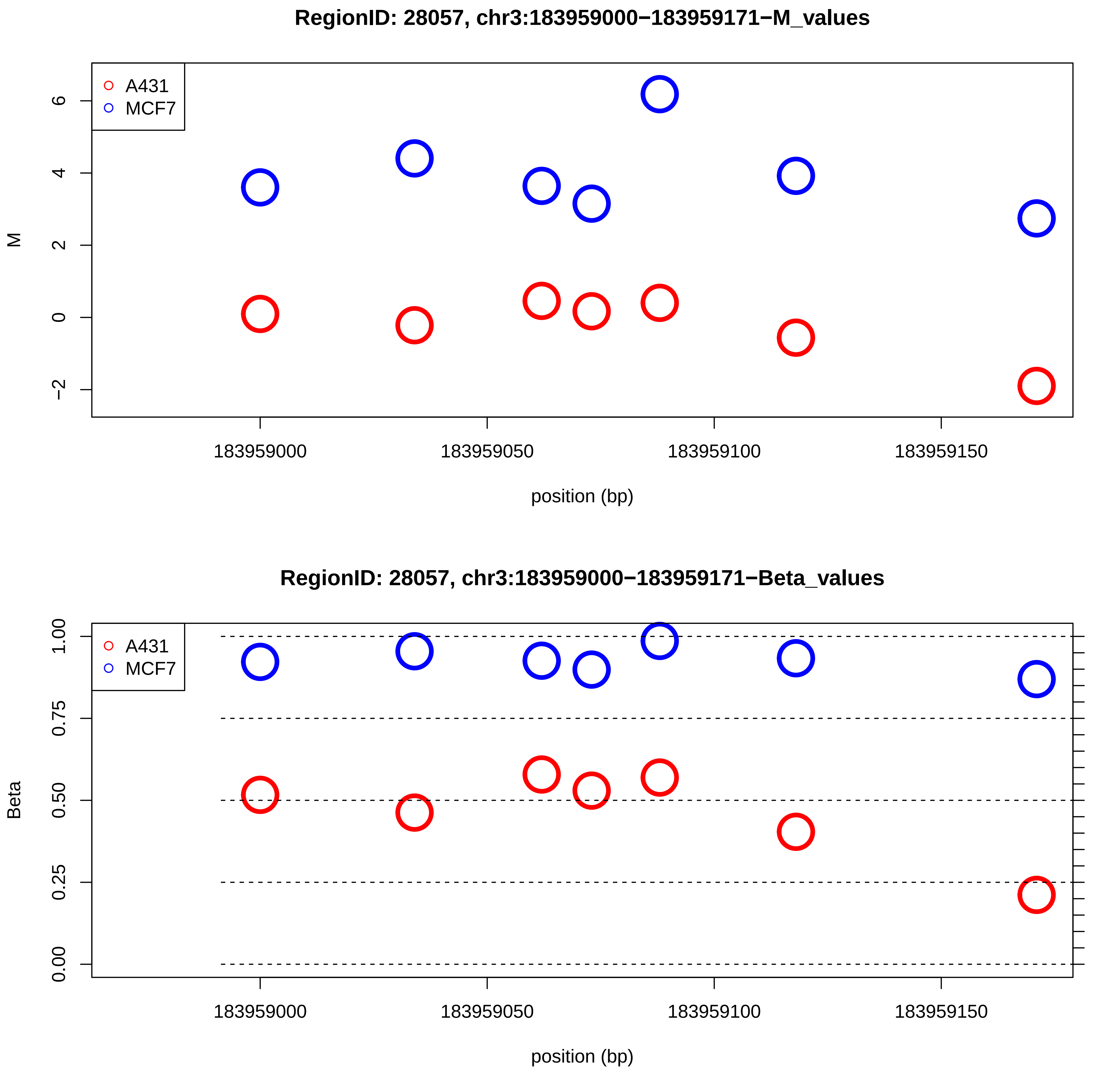
RegionID: 28057, chr3:183959000−183959171−M_values
A431
MCF7
M
position (bp)
RegionID: 28057, chr3:183959000−183959171−Beta_values
A431
MCF7
Beta
position (bp)

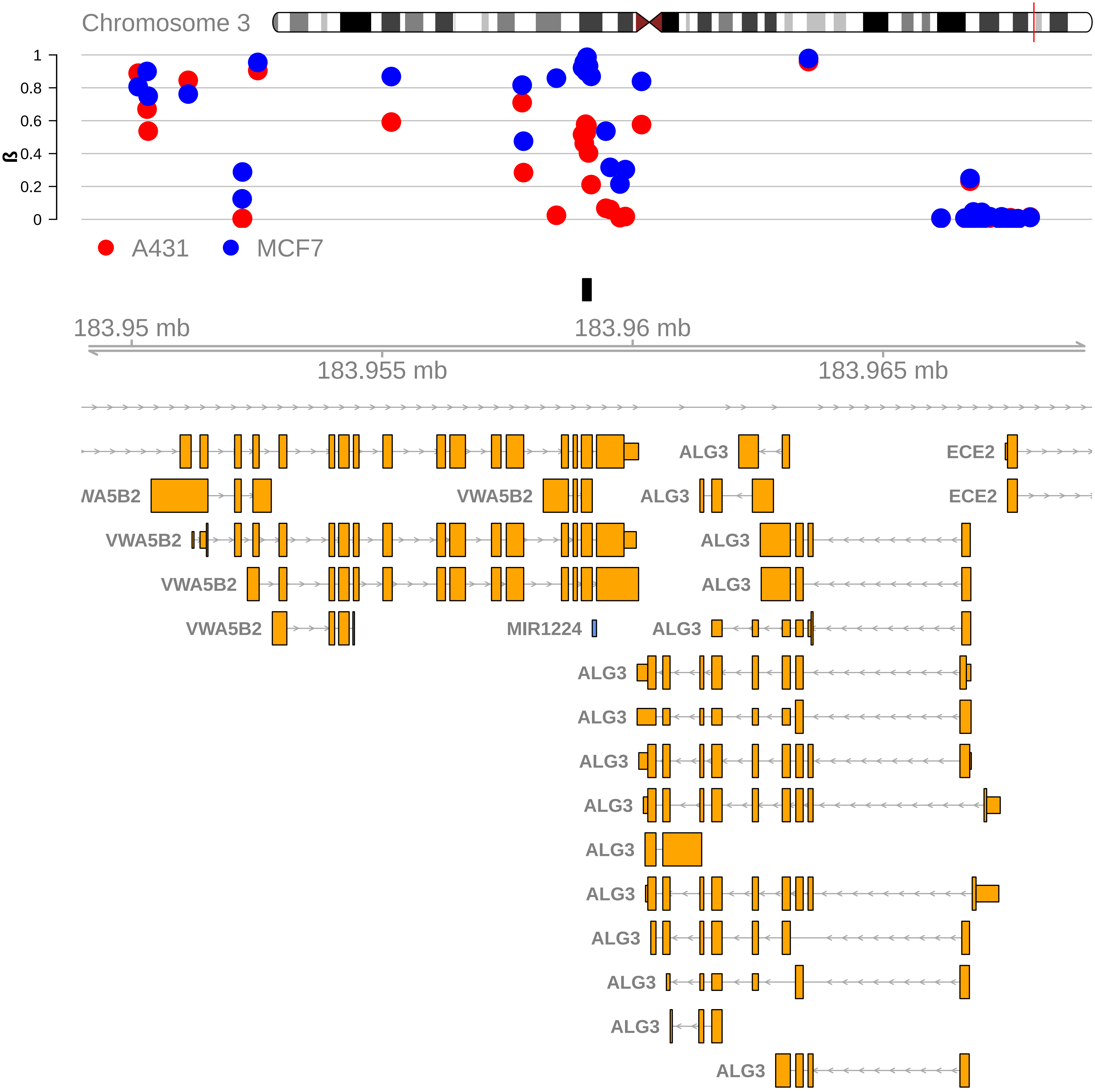

Chromosome 3
1
0.8
0.6
ß 0.4
0.2
0
A431
MCF7
183.95 mb
183.96 mb
183.955 mb
183.965 mb
VWA5B2
ALG3
ECE2
MIR1224
